# Analyzing factors that influence the folk use and phytonomy of 18 medicinal plants in Navarra

**DOI:** 10.1186/1746-4269-3-16

**Published:** 2007-04-13

**Authors:** Silvia Akerreta, Rita Yolanda Cavero, Víctor López, María Isabel Calvo

**Affiliations:** 1 Departamento de Biología Vegetal (Sección Botánica). Universidad de Navarra. C/Irunlarrea s/n, Pamplona. 31080. Navarra. Spain; 2 Departamento de Farmacia y Tecnología Farmacéutica (Sección Farmacognosia). Universidad de Navarra. C/Irunlarrea s/n, Pamplona. 31080. Navarra. Spain

## Abstract

**Background:**

This article analyzes whether the distribution or area of use of 18 medicinal plants is influenced by ecological and cultural factors which might account for their traditional use and/or phytonymy in Navarra.

This discussion may be helpful for comparative studies, touching as it does on other ethnopharmacological issues: a) which cultural and ecological factors affect the selection of medicinal plants; b) substitutions of medicinal plants in popular medicine; c) the relation between local nomenclature and uses. To analyze these questions, this paper presents an example of a species used for digestive disorders (tea and camomile: *Jasonia glutinosa, J. tuberosa, Sideritis hyssopifolia, Bidens aurea, Chamaemelum nobile*, *Santolina chamaecyparissus*...), high blood pressure (*Rhamnus alaternus*, *Olea europaea..*.) or skin diseases (*Hylotelephium maximum, H. telephium*, *Anagallis arvensis*, *A. foemina*).

**Methods:**

Fieldwork began on January 2004 and continued until December 2006. During that time we interviewed 505 informants in 218 locations in Navarra. Information was collected using semi-structured ethnobotanical interviews, and we subsequently made maps using Arc-View 8.0 program to determine the area of use of each taxon. Each map was then compared with the bioclimatic and linguistic map of Navarra, using the soil and ethnographic data for the region, and with other ethnobotanical and ethnopharmacological studies carried out in Europe.

**Results:**

The results clearly show that ecological and cultural factors influence the selection of medicinal plants in this region. Climate and substrate are the most important ecological factors that influence the distribution and abundance of plants, which are the biological factors that affect medicinal plant selection.

**Conclusion:**

The study of edaphological and climatological factors, on the one hand, and culture, on the other, can help us to understand why a plant is replaced by another one for the same purposes, either in the same or in a different area. In many cases, the cultural factor means that the use of a species is more widespread than its ecological distribution. This may also explain the presence of synonyms and polysemies which are useful for discussing ethnopharmacological data.

## 1. Background

Ethnobotany is the science that describes the relationship between humans and plants but the factors that influence that relationship are not well defined. The interdisciplinary approach is well known in ethnobotany [1-3] and can help us to understand what factors may interact and have a decisive influence on the selection of plants used by humans, and on the origin of synonyms (different names to refer to one plant taxon) [4,5] and polysemies (use of one name to refer to different plant taxa) [4,5] in the same area.

Many abiotic factors affect the distribution and abundance of plants, including climate, substrate, geomorphology, altitude and solar exposure. As suggested by Johns *et al*. [6], if a species is more abundant in one area, it will most probably be used to treat a common problem in that community. But is abundance the only factor, or are other issues involved, such as cultural influences or fashions?.

After a preliminary sampling of the study area, a bioclimatological and edaphological study of a specific location can provide an idea, *a priori*, of the most common plants that can be found there and can help to provide an overview of the potential distribution of use (meaning the same use in a different place) of the common species. Another decisive factor in the distribution of use of a species or its selection as medicinal is the history and culture of the local population, which is also affected by the degree of isolation from other cultures.

When we carried out part of the ethnobotanical study of Navarra [7], we noted striking distributions of use or phytonymies, or even possible substitutions between species according to the area in question. In the present paper, we analyze the possible influence of environmental and cutural factors in the variations and/or substitutions of 18 species, given the great diversity of the region and its exemplary nature as regards biotic and abiotic factors.

The objectives of this study were therefore:

### Objectives

(1) To demonstrate whether ecological and cultural factors have a decisive influence on the extent of use or selection and/or phytonymy of 18 medicinal plants in Navarra.

(2) To analyze and discuss which causes or factors lead to the replacement of medicinal plants in traditional medicine.

(3) To analyze the relationship between phytonymy and plant use.

### Study area

**Figure 1:**
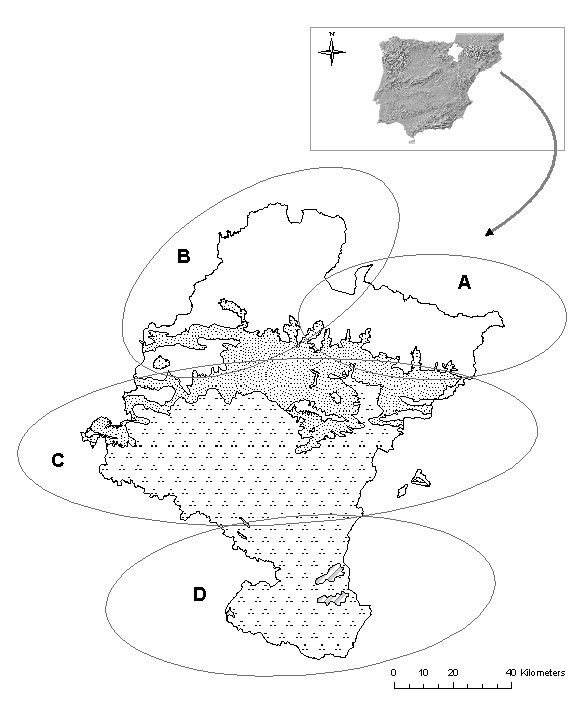
Location of Navarra, bioclimates and principal biogeographical regions. **Bioclimates**. Blank area: Temperate oceanic. Randomdotted area: Temperate oceanic sub-mediterranean. Aggregately dottedarea: Mediterranean pluviseasonal. Striped area: Mediterranean xeric. **Biogeographical areas of Navarra**. **A**-Pyrenees area. **B**-Humid area of northwest. **C**-The Central zone. **D**-The Ribera.

Navarra is a region (10.421 km^2^) in the northern Iberian Peninsula (Fig. 1) in the western sector of the Pyrenees Mountains. Starting at the limestone Pyrenean foothills reaching heights of 2,428 meters which tower over steep-sided valleys composed of flysch [8], the landscape then softens into a geomorphology based on marls and limestone marls. In the middle zone, characterized by its transitional nature, marls and limestone blend with sandstones, gradually yielding to gypsum, gravel, sand and mud which predominate in broad, windswept plains that have been eroded over the course of time.

According to López *et al*. [[9]; p90], Navarra includes two macro-bioclimates, Temperate and Mediterranean, separated by a dividing chain of mountains (Fig. 1).

The oceanic temperate bioclimate appears in the northern part of the territory, and is characterized by mild temperatures and high precipitation throughout the year. More to the south, as precipitation decreases, the oceanic temperate bioclimate changes to the sub-Mediterranean variant and finally to the seasonal-rainfall Mediterranean bioclimate, characterized by seasonal drought (favoring species that are specifically Mediterranean). The latter bioclimate occupies most of the territory to the south, although there is also a small island of xeric Mediterranean in an area that is now a Biosphere Reserve due to its ecological importance (Bardenas Reales) [10]. Owing to its geological, geomorphological and bioclimatic variability, it has a rich flora (2650 vascular plants) [11].

All these climatic factors, along with the geomorphologic and edaphological ones, which change sharply in a relatively small area, provide a great diversity of plant communities, such as the forests of elm trees, both basophilic and acidophilic (one of the best conserved in Europe is the *Irati *forest), the groves of *Quercus robur *L. oak and *Q. humilis *Miller, as well as holm oak, evergreen oak, gall oak, and Pyrenean pastures rich in the Alpine and sub-Alpine plant series, among others [12,13].

**Figure 2:**
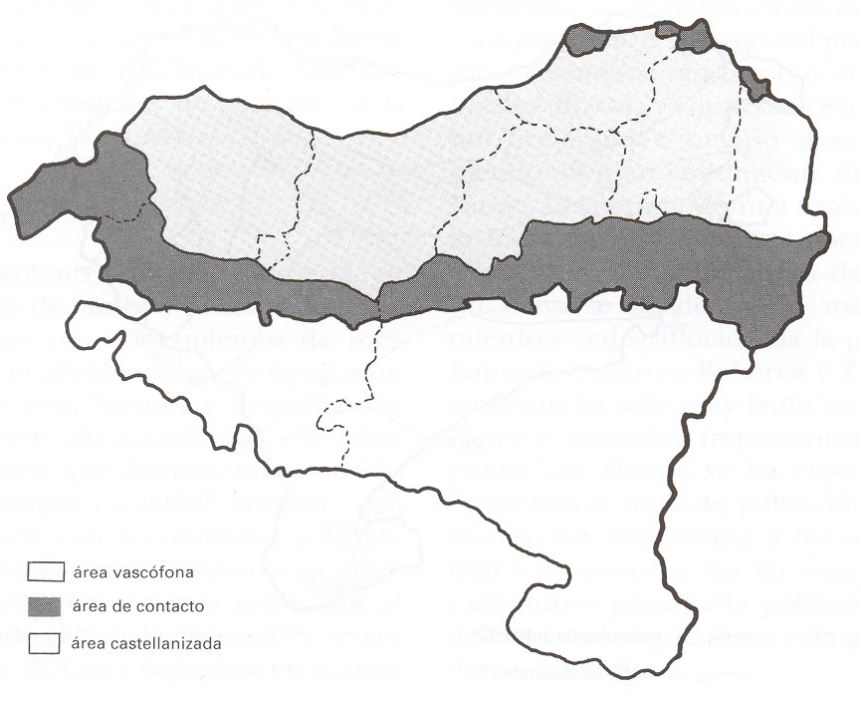
Linguistics areas of Vasconia (From "Atlas Etnografico de Vasconia. Medicina popular en Vasconia" [14]). Blank area: Spanish-speaking area. Light grey: Contact area. Dark grey: Basque-speaking area.

The area was inhabited by the Basques until the arrival of the Romans. It was later populated by the Visigoths. Nowadays, only 10% of the population of Navarra speaks "Euskera", the language of the Basque ethnic group, though the percentage of Euskera speakers was higher in recent times (s. XVIII-XIX) [14], which influences some aspects of present-day culture, including ethnobotany. Nonetheless, in the north-west there is a greater influence of the Basque culture and language, which has a direct influence on the use and names of the plants (Fig. 2).

## 2. Material and methods

Field and laboratory work began on January 2004 and lasted until December 2006. During that time we interviewed 505 informants in 218 locations in Navarra (Fig. 1). Information was collected using semi-structured ethnobotanical interviews as described by Martin [14]. Those interviews were always carried out spontaneously with people born or having lived most of their lives in the region studied.

Of those interviewed, 54.3% were women. The ages of the interviewees ranged from 22 to 100 year, the age group from 71 to 80 years representing the mean. When possible, the conversations were recorded.

In the field work we noted for each species the local name, place and collection method, drying and preservation system, parts or organs used and method of preparation, dosage and administration. The species were collected with the interviewee *in situ *and then identified in the laboratory using keys for botanical determination, labeled and included in the PAMP Herbarium at the School of Science. The bibliography used for taxonomic determination was *Flora Iberica *[16], and for unpublished families, we used *Flora of the Basque Country *[17]. The local names were transcribed and processed following the methodology of phytonymy [18].

From the preliminary ethnobotanical study, 18 species were selected for the present study, on the following grounds:

1-species used for 3 of the commonest disorders in Navarra [7]: gastrointestinal, circulatory (hypertension) and dermatological complaints.

2-From these, we selected those species which, in addition to being mentioned by more than three informants, were found to have: a) noteworthy distributions or areas of use (densely within one area, or across the whole region), and/or clear variations in phytonymy according to area.

For each of these species we drew up a map of the distribution of use and/or phytonymy using the program Arc-View 8.0, taking the coordinates of the places where the species were mentioned by the informants. These maps were compared with the bioclimatic [9] and linguistic maps of Navarra (Fig. 2) [13]; edaphological data about the region [8]; ethnographic research carried out in the study area, such as that by Lacoizqueta [19], Barriola [20], Erkoreka [21] or Etniker-Euskalerria [14], among others cited in the text; ethnobotanical studies performed in different regions of the Iberian Peninsula, such as Bonet *et al*. [22-24], Agelet and Vallès [25-27], Fajardo *et al*. [28], González-Tejero *et al*. [29], Lastra and Bachiller [30], Martínez *et al*. [31], Vázquez *et al*. [32], Pardo de Santayana *et al*. [33], Villar *et al*. [34], Tardío *et al*. [35], or Parada *et al*. [36], as well as other ethnobotanical and pharmaceutical studies performed in Europe and in the rest of the world (cited in text).

## 3. Results and discussion

The selected species used for improving the digestion, high blood pressure and for skin disorders are presented in Table 1, 2 and 3. All the species collected are still used for those purposes.

**Table 1:** Selected species used for gastrointestinal disorders and called "té" *(tea) *or "manzanilla/kamamila" (camomile) in Navarra

	**Species**	**Habitat**	**Local name**		**PU**^**a**^	**P**^**b**^	**Popular use**	**C**^**c**^
			***Spanish***	***Basque***				
**Medicinal plants named "té"**								
	**ASTERACEAE**							
1	*Bidens aurea *(Aiton) Sherff (PAMP 19668–19675)	Cultivated	Té Americano, té del fraile		AP	I	Stomach ache.	8
2	*Jasonia glutinosa *(L.) DC (PAMP 18642, 18768–18771)	Cracks, rock ledges and fissures	Té de roca, té de peña		AP	I	Digestive, stomach ache, to purify the blood and Raise the spirits, clear the mind.	113
3	*Jasonia tuberosa *(L.) DC (PAMP 18772, 18773)	Marly substrate	Té		AP	I	Stomach ache and to Raise the spirits.	34
	**LAMIACEAE**							
4	*Sideritis hyssopifolia *L. subsp. *guillonii *(Timb.-Lagr.) Rouy (PAMP 18698, 18699, 18750, 18751)	Cracks, rock ledges and fissures	Té de roca		AP	I	Digestive, stomach ache and to bloating.	5

**Medicinal plants named "kamamila or manzanilla"**								
	**ASTERACEAE**							
1	*Anthemis arvensis *L. (PAMP 18758)	**RUDERAL**	Manzanilla basta		FT	I	Digestive, stomach acidity, gut pain.	8
2	*Chamaemelum nobile *(L.) All. (PAMP 18640, 18641, 18759–18763)	Temperatura pasture	Manzanilla, manzanilla fina, manzanilla de monte, manzanilla de Urbasa, Aralar	Kamamila	FT	I	Digestive, gut pain, for bad mood, stomach acidity, laxative, anti-diarrhoeal, tranquillizer.	119
3	*Helichrysum stoechas *(L.) Moench subsp.*stoechas *(PAMP 18766, 18767)	Dry or stony pastures	Manzanilla, manzanilla dulce, manzanilla fina		FT	I	Digestive	15
4	*Matricaria recutita *L. (PAMP 18764)	Cultivated	Manzanilla		FT	I	Stomach ache	5
5	*Santolina chamaecyparissus *L. subsp. *squarrosa *(DC) Nyman (PAMP 18643–18649, 18749, 18775–18782)	Open scrubland and dry or stones pastures	Manzanilla, manzanilla de monte, manzanilla basta, manzanilla de burro, hierba para el mal de las gallinas		FT	I	Digestive, headache, stomach ache, belly pain, stomach problems, to clean the blood (depurative), tranquillizer nuisance and veterinary (digestive for sheeps).	169
						HB (D)	Woman hygiene.	
6	*Tanacetum parthenium *(L.) Sch. Bip. (PAMP 18650, 18783, 18784)	Cultivated	Manzanilla de huerta, manzanilla amarga	San Juan Lorek	FT	I	Digestive, stomach ache, bile problems.	8
						HB (D)	Woman hygiene.	
						R	To protect the country house from storms and misfortune.	10

a) PU: part used; AP: aerial part; FT: floral top

b) D: decoction; HB: hip bath; I: infusion; R: rite; V: veterinary use.

c) C: Citations.

**Table 2:** Selected species used for circulatory disorders (high blood preassure).

	**Species**	**Habitat**	**Local name**		**PU**^**a**^	**P**^**b**^	**Popular use**	**Citations**
			***Spanish***	***Basque***				
	**OLEACEAE**							
1	*Olea europaea *L. subsp. *europaea*	Cultivated	Olivo		L	D	High blood preassure	33
	**RHAMNACEAE**							
2	*Rhamnus alaternus *L. (PAMP 19664–19667)	Woods and scrublands		Karraskila	B	D	High blood preassure	15
	**URTICACEAE**							
3	*Urtica dioica *L. (PAMP 19652–19661)	Ruderal	Ortiga	Atsun, osina	AP	I	High blood preassure	18
4	*Urtica urens *L. (PAMP 19662)	Ruderal	Ortiga	Atsun, osina	AP	I	High blood preassure	

a) AP: aerial part; B: branch; L: leaf.

b) D: decoction; I: infusion.

**Table 3:** Selected species used for skin disorders.

	**Species**	**Habitat**	**Local name**		**PU**^**a**^	**P**^**b**^	**Popular use**	**Citations**
			***Spanish***	***Basque***				
	**CRASSULACEAE**							
1	*Hylotelephium maximum *(L.) Holub. (PAMP 19676, 19678–19684)	Cultivated	Curalotodo		L	DA	Skin diseases:wounds, spots...	23
2	*Hylotelephium telephium *(L.) H. Ohba (PAMP 19677)	Cultivated	Curalotodo		L	DA	Skin diseases:wounds, spots...	1
	**PRIMULACEAE**							
3	*Anagallis arvensis *L. (PAMP 19647–19649)	Ruderal		Pasmobelarra	AP	P, O	Skin diseases: infected wounds ("bixiko")	15
4	*Anagallis foemina *Mill. (PAMP 19650)	Ruderal		Pasmobelarra	AP	P, O	Skin diseases: infected wounds ("bixiko")	3
	**LILIACEAE**							
5	*Allium cepa L*.	Cultivated	Cebolla	Tipula	L	O, DA	Skin diseases: infected wounds ("bixiko", "panadizo"..)	82

a) AP: aerial part; L: leaf

b) DA: direct application; P: poultice; O: ointment

**Figure 3:**
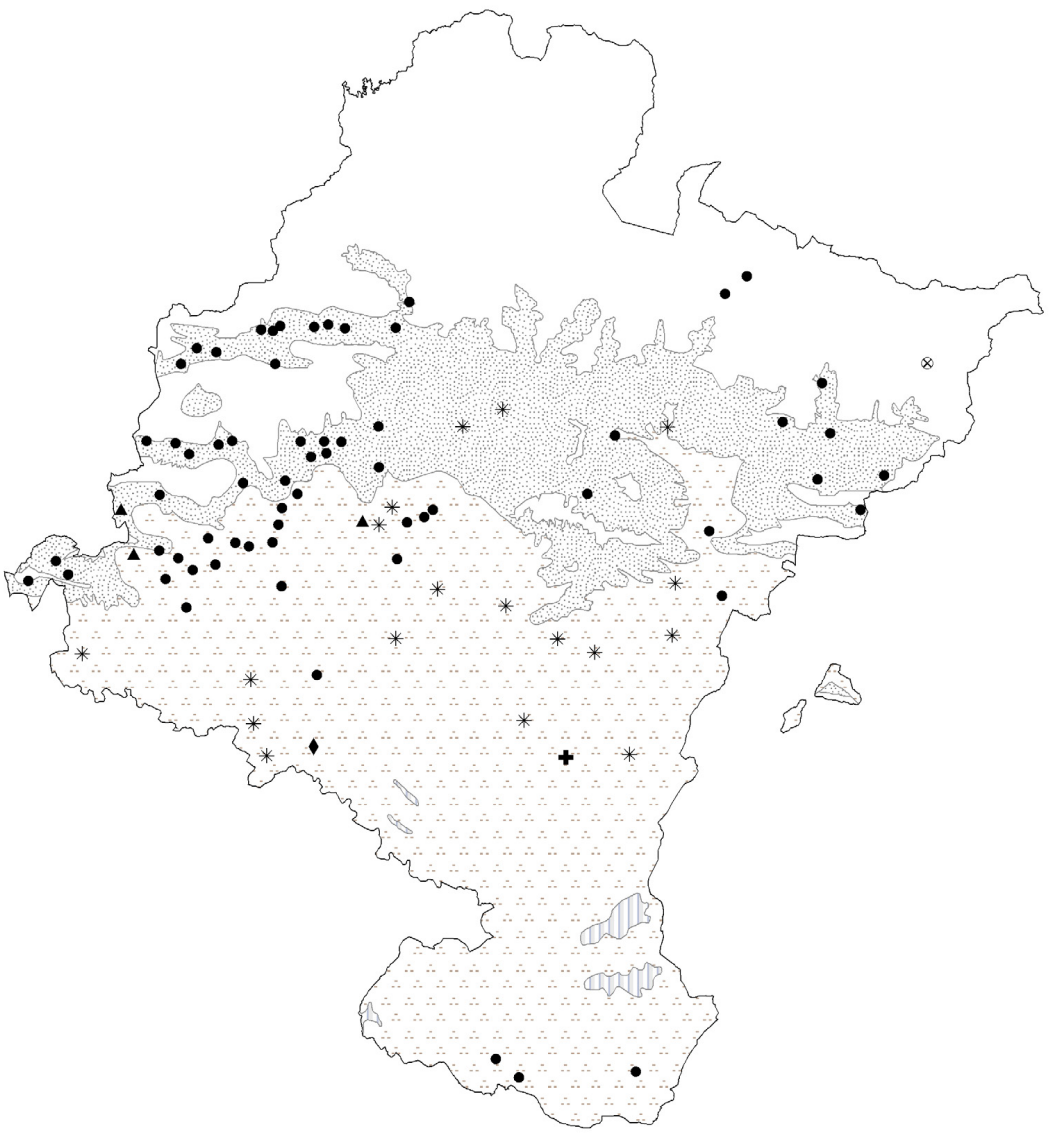
Map of the distribution of use of teas. ● *Jasonia glutinosa*.  *Jasonia tuberosa*. ▲ *Jasonia glutinosa *and/or *Jasonia tuberosa*. + *Jasonia tuberosa *and/or *Bidens aurea*. ◆ *Jasonia glutinosa *and/or *Bidens aurea*. ⊕ *Sideritis hyssopifolia *subsp.*guillonii*. **Bioclimates**. Blank area: Temperate oceanic. Random dotted area: Temperate oceanic sub-mediterranean. Aggregately dotted area: Mediterranean pluviseasonal. Striped area: Mediterranean xeric.

Teas and chamomiles for digestive remedies:

Among those used for digestive problems, there are two groups of species: those with names derived from "té" (tea) and those from "manzanilla" or "kamamila" (chamomile). *Jasonia glutinosa *or "té de roca" (rock tea) is the species called tea which is most commonly used in Navarra (see Fig. 3). It is mostly used by inhabitants close to calcareous mountain ranges, as is the case in other studies where that species has been cited [33-35,37-40]. That is because rock tea has an exclusive habitat in cracks, rock ledges and fissures that receive plenty of sunlight, thus its area of use is first influenced by the substrate. However, people that live in communities of the south of Navarra far from the areas where it grows commented that it should be harvested in the mountains, and that in the past, it could be purchased in the villages. We therefore believe that the cultural factor, rather than the environmental factor, determines its distribution. Previous studies have shown that its chemical composition includes many oxygenated terpenes and sesquiterpene derivatives [41], the latter of which have anti-inflammatory effects *in vitro *[42]. There are also a considerable amount of saponins, both steroidal and triterpenic [33], as well as tannins [43], which could explain its use, albeit a limited one, as an anti-diarrheic. Because of these chemical properties, or because the influence of culture or fashion, the use of this species has spread beyond its natural boundaries (Table 4).

**Table 4:** Relation between use distribution and ecological distribution^a^.

***Species***	**UD > ED**	**UD ± = ED**	**UD< ED**
*Allium cepa*	•		
*Anagallis arvensis-A. foemina*			•
*Anthemis arvensis*			•
*Bidens aurea*	•		
*Chamaemelum nobile*	•		
*Helichrysum stoechas*			•
*Hylotelephium maximum-H. telephium*	•		
*Jasonia glutinosa*	•		
*Jasonia tuberosa*			•
*Matricaria recutita*	•		
*Olea europaea*	•		
*Rhamnus alaternus*			•
*Santolina chamaecyparissus*			•
*Sideritis hyssopifolia*			•
*Tanacetum parthenium*	•		
*Urtica dioica-U. urens*		•	

a) UD: use distribution; ED: ecological distribution.

Another species called and consumed as té (tea) is *Jasonia tuberosa*, which is used in the south, in places where the use of *Jasonia glutinosa *declines, which indicates that the use of one replaces that of the other. It only grows in flat locations with a marl substrate and Mediterranean environment [8,17]. Thus, the substrate and climate are decisive for the distribution of use of that species and help to explain this polysemy and substitution: the same use and same name indicate that if *Jasonia glutinosa *is not present in the area, but a similar one, in this case *Jasonia tuberosa*, is available, then it is replaced by the latter. When an informant in this area goes north to obtain *Jasonia glutinosa*, he or she considers this species to be of top quality, while *Jasonia tuberosa *is of secondary quality and should only be used as a substitute. The aerial parts of this European endemism, which is only found in the Iberian Peninsula and southern France [39], are also used as a digestive and to clear the mind. According to Gil Pinilla [44], its main component, camphor, is used to treat gastrointestinal disorders, and has spasmolytic, anesthesic, analgesic, antiemetic, antiseptic and carminative properties.

*Sideritis hyssopifolia *L. ssp. *guillonii*, also called "té de roca" (rock tea), is considered endemic to the northwest Mediterranean-orophyte [45,46]. Several species of *Sideritis *are used in different European regions [24,25,34,35,37,47-52]. This polymorphic taxon appears, as its colloquial name indicates, in rocky outcrops or crevices in high crags, often in the same habitat as *Jasonia glutinosa*; however, in Navarra the only population of *Sideritis hyssopifolia *has been mentioned in an isolated valley of glacial origin, near the mountain peaks. In this isolated place it is the cultural factor which has kept this species in use into our own day; this is also the case in the western mountains, where the cultural factor is the deciding one, though in this case it favors the exclusive use of *Jasonia glutinosa*, as we saw above. This means that *Sideritis hysopifolia *is less widely used than its geographical distribution might indicate (Table 4). Since there are different species of *Sideritis*, scientific research on that genus is quite diverse as well. However, the chemical composition in broad outline of *S. hyssopifolia *is known: Rodriguez Lyon *et al*. [53] have shown that its digestive properties are due to flavonoids.

*Bidens aurea *is a tea which is known today only in a few places in the south of Navarra. A native of Central America [54], it was brought back by a missionary friar many decades ago, according to our interview data. It used to be grown, but now occurs as an invader in irrigation channels, as Pardo de Santayana & Morales state for other regions within the Iberian peninsula [33]. Its cultivation and naturalization, favored by environmental factors, are exclusively explained by cultural factors supported by the chemical properties of the plant [33,55]. As it is not autochthonous and has to be cultivated, its distribution of use is greater than its ecological distribution.

None of the four species known as tea in Navarra have a monograph in WHO [56], ESCOP [57] or the E Commission [58]. This is most probably due to the lack of research on their chemical composition and, according to Muñoz-Centeno [59], since these medicinal species are only used in the Iberian Peninsula, they are not very well analyzed, although Fernández [60] states that the fact that they are still used confirms their effectiveness.

**Figure 4:**
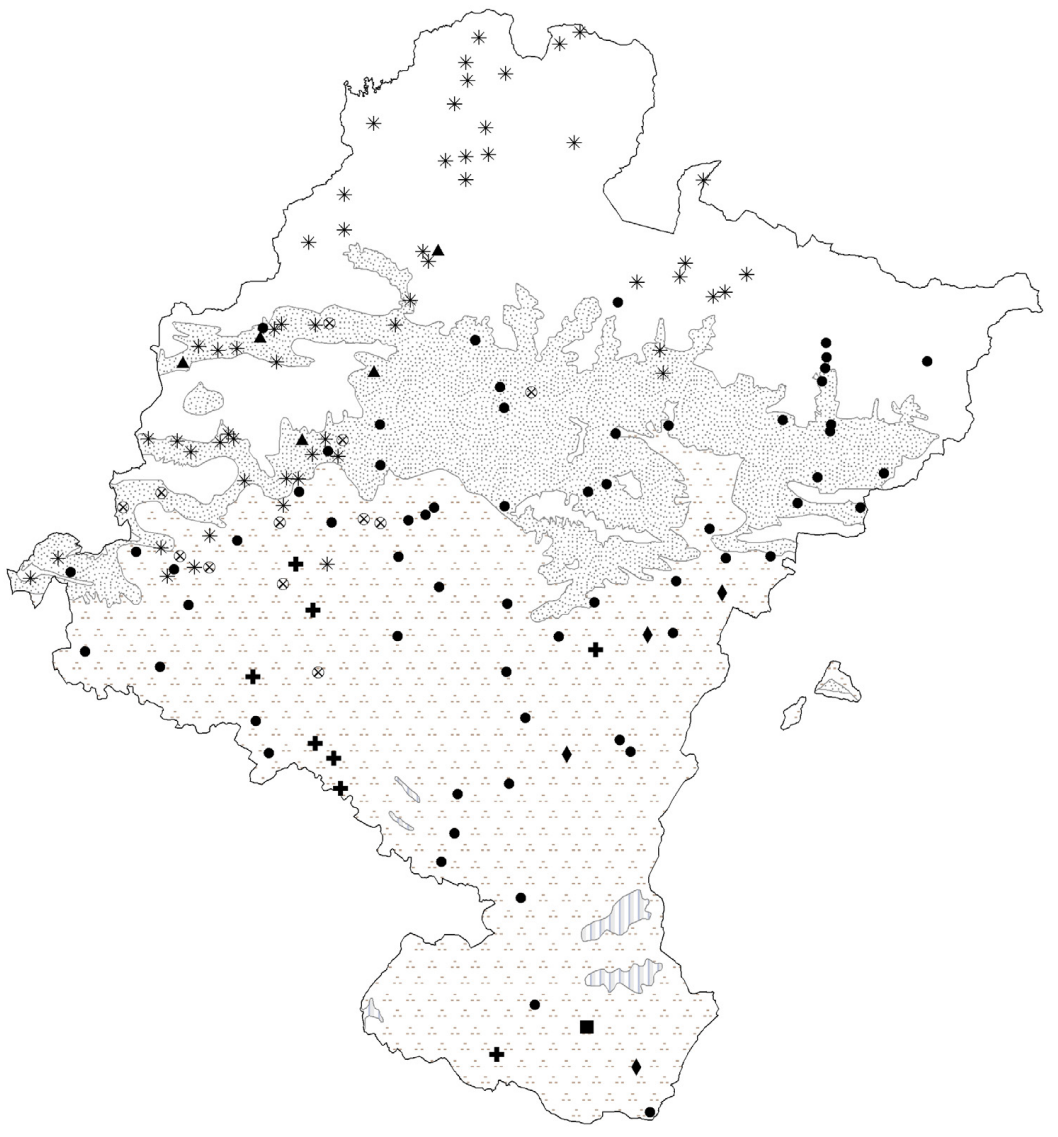
Map of the distribution of use of camomiles (except *Tanacetum parthenium*). ● *Santolina chamaecyparissus *subsp. *squarrosa*. *Chamaemelum nobile*. ⊕*Chamaemelum nobile*. and/or *S. chamaecyparissus *subsp. *squarrosa*. ▲ *Chamaemelum nobile *and/or *Anthemis arvensis*. + *Santolina chamaecyparissus *subsp. *squarrosa *and/or *Helichrysum stoechas*. ◆ *Santolina chamaecyparissus *subsp. *squarrosa *and/or *Matricaria recutita*. ■ *Helichrysum stoechas*. **Bioclimates**. Blank area: Temperate oceanic. Random dotted area: Temperate oceanic sub-mediterranean. Aggregately dotted area: Mediterranean pluviseasonal. Striped area: Mediterranean xeric.

**Figure 5:**
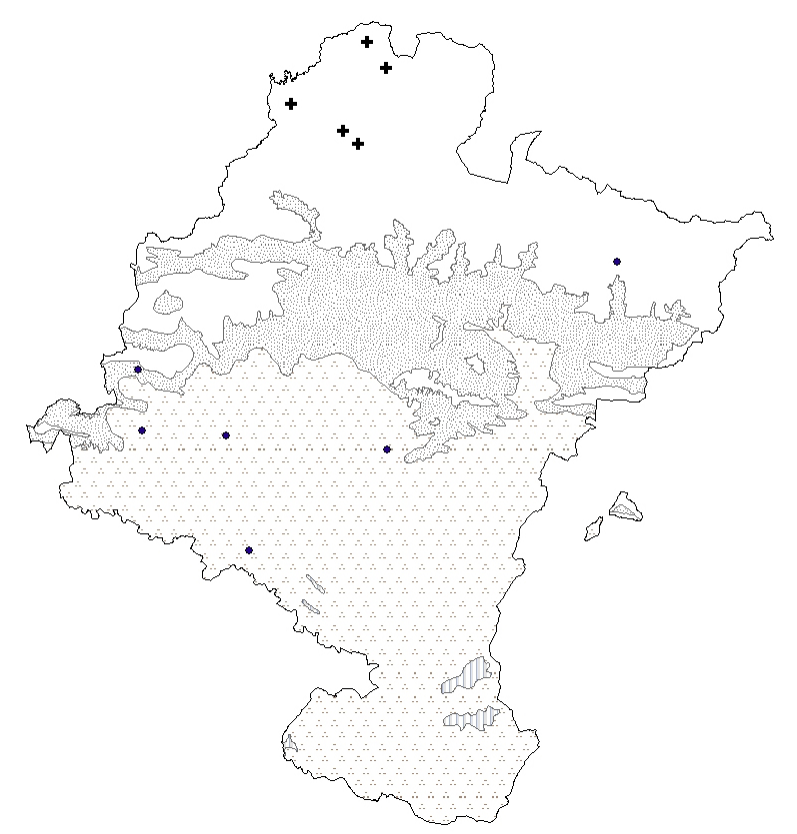
Map of the distribution of use of *Tanacetum parthenium*. + Like as "San Juan Lorek". ● Like as camomile. **Bioclimates**. Blank area: Temperate oceanic. Random dotted area: Temperate oceanic sub-mediterranean. Aggregately dotted area: Mediterranean pluviseasonal. Striped area: Mediterranean xeric.

Other herbs used to treat digestive disorders include many species known as chamomile (Fig. 4 and Fig. 5). All these species belong to the *Asteraceae *family and have similar indications (Table 1). Popularly in Navarra there has always been some confusion between the stomach and the intestines, since everything in the abdomen is considered to be the same thing, including the abdominal viscera. As a result, stomach or intestinal pains, or even specific female pains (e.g., menstrual pain), sometimes have similar remedies [14].

*Santolina chamaecyparissus *subsp. *squarrosa *was found to be the most commonly used plant called "manzanilla" (chamomile), which is at variance with the assertion by García Bona [61] that it was "rarely" used in Navarra. It is characteristic of Mediterranean climates with a basophilic soil, and is distributed throughout the Mediterranean bioclimate and Temperate Oceanic of sub-Mediterranean variant and, in this case, on sunny slopes, as is the case in other regions with similar bioclimatic characteristics [22,24,34,40,62-64]. As a result of its ecological distribution, it is more frequent in the central zone of Navarra. It is interesting that although it is the most often mentioned chamomile, it is also the one which has pejorative names when its use overlaps with *Chamaemelum nobile *or *Helichrysum stoechas*, a phenomenon which points to the fact that it is often replaced by these species.

*Chamaemelum nobile *(L.) All. follows *Santolina chamaecyparissus *subsp. *squarrosa *in terms of use. However, this use is distributed across marl-based pastures throughout the Temperate oceanic climate, that is, in northern Navarra. The use of this chamomile has also been noted in other ethnobotanical studies in areas that have a similar temperate climate to northern Navarra [65-69]. However, it is also used in the Mediterranean region by locals who go to the northern mountains for this special chamomile. Interestingly, these people that go to the northern areas to gather *Chamaemelum nobile*, call *Santolina chamaecyparissus *by the pejorative name "manzanilla basta" (rough or donkey chamomile) o "hierba para el mal de las gallinas" (grass for hen sickness), indicating that *S. chamaecyparissus *is a substitute for *C. nobile*. Its chemical composition and pharmacological activity are very similar to *Matricaria recutita *[70]. The apigenin derivatives in its chemical composition may be responsible for its spasmolytic activity, which explains its digestive and sedative properties [71]. These mean that it is regarded culturally as a first-class chamomile, thereby accounting for the fact that it is used beyond the boundaries of its ecological distribution.

*Helichrysum stoechas *is used as a chamomile in the South, in the Mediterranean area with poor soils [18]. It has also been cited in other warmer locations in the Iberian Peninsula [62,63] and in Navarra it has almost the same ecological distribution as *S. chamaecyparissus *[17], which are known where they are used as "manzanilla fina" ("fine chamomile") and "manzanilla basta" ("rough chamomile") respectively. The cultural factor, supported by the plant's chemical properties [72,73], was not decisive enough to prevent its distribution of use being smaller than that of *S. chamaecyparissus *(which was favored by environmental factors which make it much more widely available).

*Anthemis arvensis *L. or "manzanilla basta " (rough chamomile) has been cited in the same humid area as *Chamaemelum nobile *and, while the latter is harvested in the green pastures in the mountains, *A. arvensis *appears close to houses and, according to informants, is used for emergencies, when *C. nobile *is not available. In fact, according to Ladero *et al*., [62: 184], this species is often used as an adulteration of *C. nobile *and *Matricaria recutita*. In this case, its wide availability does not mean that it is used more, since it is only used sporadically, and so the pattern proposed by Johns *et al*. [6] is not followed. In fact, the cultural factor is decisive in this case, because this species is considered to be of second-rate quality, and it is therefore regarded as a mere substitute for *C. nobile*, which has superior chemical properties that make it the plant of choice.

Another species called chamomile is *Tanacetum parthenium*. In Navarra there is a synonymy for this plant, explained by its two different uses: in the northeast and in the Basque-speaking area, the aerial parts of the plant called "San Juan Lorek" are collected in a bunch and placed on the door of country houses to protect them from "lightning and misfortune". According to Abella [74: 276], this use has also been noted in villages of Vizcaya (Basque Country). On the other hand, it is cultivated to a lesser extent in the orchards of some villages to obtain a infusion until the so called "manzanilla silvestre" (wild chamomile, *S. chamaecyparissus *subsp. *squarrosa*) is available in other areas (that is, warmer areas than the Pyrenean villages in question). As it is more widely available, cultural factors mean that it is used as a substitute for the proper chamomile (*S. chamaecyparissus*) when this is not available or accessible. Its dried aerial flower parts are consumed as a digestive infusion, to wash the eyes and as a hip bath for woman. These indications correspond to its chemical composition [75-78]. Nonetheless, none of these indications have yet been recognized in the monographs written by the WHO [56] or ESCOP [57] on these species.

Lastly, we found *Matricaria recutita*. It is still used (sometimes cultivated) for medicinal purposes in various areas of the Iberian Peninsula [22,35-38,40,66,68,79,80]. Nonetheless, this plant is in the group of chamomiles used least in the traditional medicine of Navarra. Cultural reasons explain why this allochthonous species is not chosen for cultivation despite the fact that it is the most popular chamomile in the world with properties that are widely described in the bibliography of the subject [81-85].

**Figure 6:**
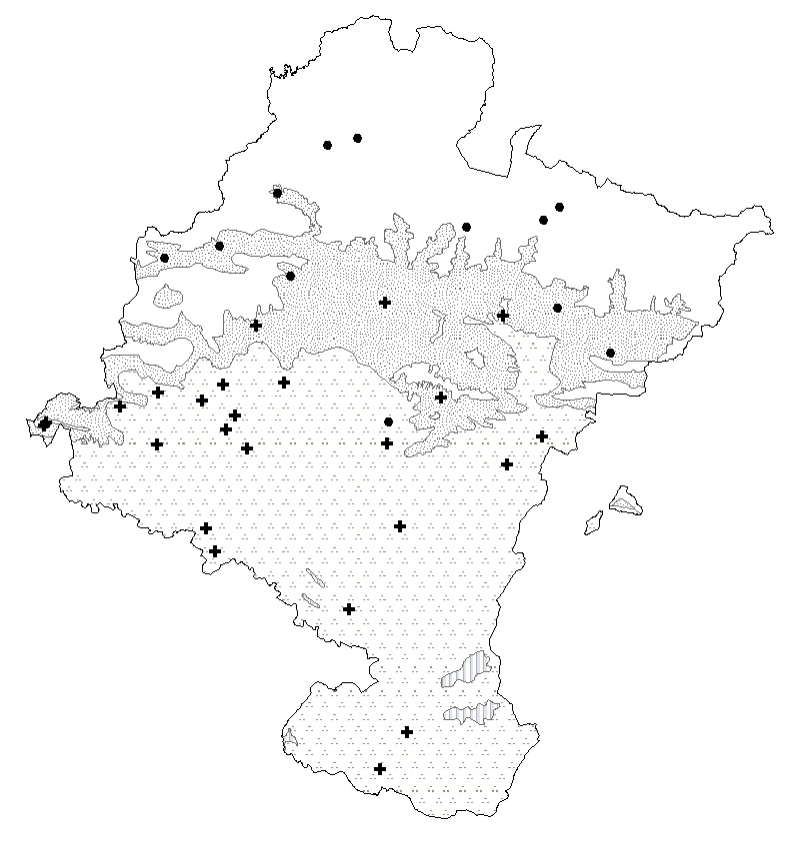
Map of the distribution of use of hypotensive species (1) (except *Urtica dioica*). + *Olea europea*. ● *Rhamnus alaternus*. **Bioclimates**. Blank area: Temperate oceanic. Random dotted area: Temperate oceanic sub-mediterranean. Aggregately dotted area: Mediterranean pluviseasonal. Striped area: Mediterranean xeric.

**Figure 7:**
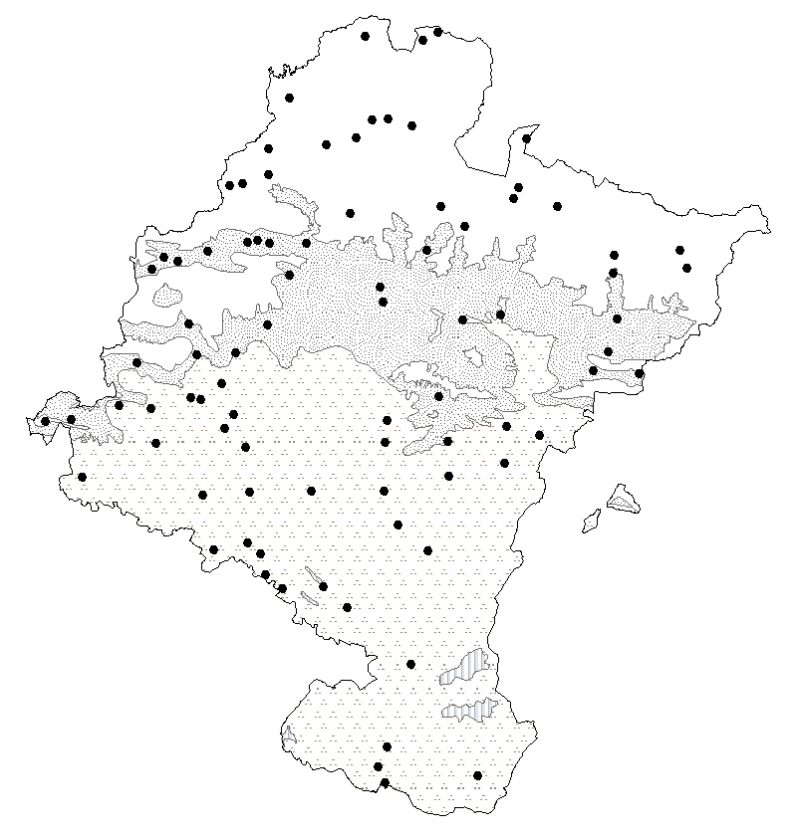
Map of the distribution of use of hypotensive species (2). ● *Urtica dioica*. **Bioclimates**. Blank area: Temperate oceanic. Random dotted area: Temperate oceanic sub-mediterranean. Aggregately dotted area: Mediterranean pluviseasonal. Striped area: Mediterranean xeric.

Circulatory disorders: plants use for high blood pressure:

*Rhamnus alaternus *or karraskila, which grows in woods and scrublands throughout the area [17,45] (Table 2), is more used in the principal Basque-speaking area, as its use depends on cultural as well as on biogeographical factors (Fig. 6). In the south, olive production was adopted a long time ago and the inhabitants learned its medicinal uses from their neighbors to the south. Indeed, such uses have been reported in many surveys carried out in the Iberian Peninsula [24,35] and its leaves have been shown to contain triterpenoids that substantiate its traditional indications [86]. Another plant in this group, which is more cosmopolitan, is "ortiga", "atsun" (of "asun" nettle, [19]) or "osina". This plant, with its similar common names and morphological characteristics, according to Linares *et al*. [87], is ruderal [17] and therefore widely available in case of need. The fact that it is not necessary to climb mountains to obtain it (as is the case with *Rhamnus alaternus*), or to grow it oneself (*Olea europaea*), accounts for its notable widespread use throughout the region (Fig. 7). Its distribution of use is the largest for plants employed to treat circulatory disorders, and is the same size as its ecological distribution.

**Figure 8:**
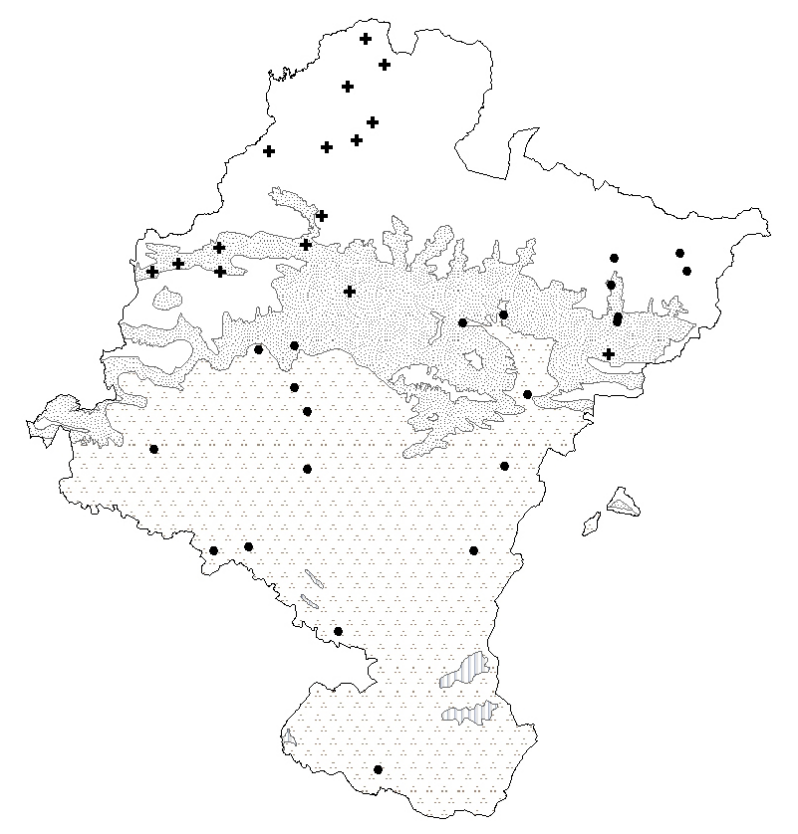
Map of the distribution of use of skin species (1) (except *Allium cepa*). + *Anagallis arvensis *and/or *Anagallis foemina*. ● *Hylotelephium maximum *or *Hylotelephium telephium*. **Bioclimates**. Blank area: Temperate oceanic. Random dotted area: Temperate oceanic sub-mediterranean. Aggregately dotted area: Mediterranean pluviseasonal. Striped area: Mediterranean xeric.

**Figure 9:**
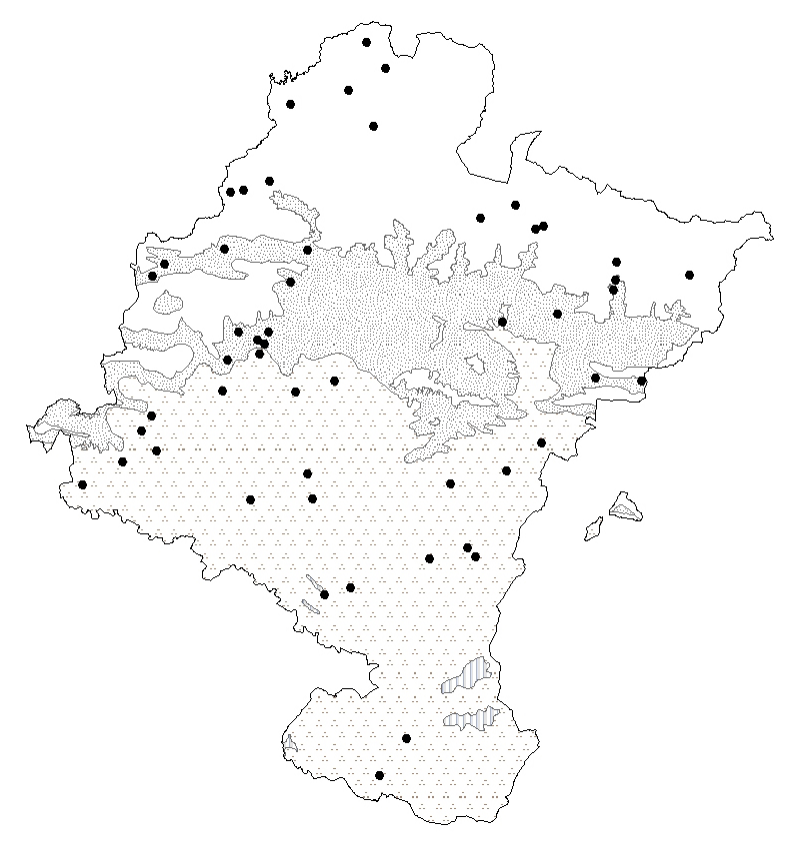
Map of the distribution of use of skin species (2). ● *Allium cepa*. **Bioclimates**. Blank area: Temperate oceanic. Random dotted area: Temperate oceanic sub-mediterranean. Aggregately dotted area: Mediterranean pluviseasonal. Striped area: Mediterranean xeric.

Skin disorders: cuts, whitlows and *bixikos *(infected spots):

The morphology of *Hylotelephium *leaves facilitate its use as a "band-aid" (Table 3): the epidermis of the fresh leaves is peeled and placed directly on the wound. This healing property was noted by John Gerard in 1597 [70]. This plant is cultivated and used for this purpose throughout most of Navarra (Fig. 8), except the linguistic Basque area, where it is substituted by the use of two wild species: *Anagallis arvensis *and *A. foemina *and called indifferently "pasmobelarra". Although those species appear to be ruderal throughout the territory of Navarra, without being affected by climate or substrate, the cultural factor determines the distribution of its use. This is a clear evidence that the dermatological use of *Hylotelephium*-*Anagallis *is a cultural substitution: use of *Anagallis *is reduced to the Basque area in Navarra, with its isolated houses where ancestral Basque traditions are still kept alive. Indeed, three people interviewed referred to those plants in the elaboration of ointments used by "pelotaris" (hand ball players) to cure wounds in the hand after matches. However, owing to its importance in Basque culture, it has been mentioned in several ethnography [14,88] and botanic [60,89] works on the region. Curiously, *Hylotelephium maximum *was cited three times in this reason for a non-dermatological application. *Hyloteolephium *is here known as "belarribelarra" (herb of the ear or eye), and the juice from its leaves is used to relieve the pain caused by cold to the ear. To continue with the plants that have dermatological applications, *Allium cepa *L. is the most widely used species. Although it is allochthonous, it is part of the staple diet of all the houses and farm communities in Navarra, and is therefore always at hand. Its availability, which is favored by the underlying cultural factor which led to its cultivation, has been decisive in ensuring widespread use (Fig. 9). Moreover, the plant's medicinal antibacterial properties support the traditional application to which it is put [58].

## 4. Conclusion

-The results clearly show that ecological and cultural factors influence the choice and phytonymy of 18 medicinal plants in Navarra.

-Climate and substrate are the most important abiotic factors that determine a plant's availability and accessibility for medicinal use: if a plant is abundant, it is more likely to be used, and vice versa. Even in the case of *Santolina chamaecyparissus *subsp. *squarrosa*, the wide availability of this species as a result of environmental factors means that it is the most widely used chamomile in Navarra, over and above the cultural elements which brand it with a pejorative phytonym and relegate it to second position in areas where its use overlaps with that of *Chamaemelum nobile *and *Helichrysum stoechas*.

-Nonetheless, the cultural factor in Navarra is decisive when it comes to the medicinal use of plants, over and above their abundance and natural availability. The cultural background determines that plants are chosen for two reasons: 1) the local population have a sense of the chemical and medicinal properties of the plant, as in the case of *Jasonia glutinosa *and *Chamaemelum nobile*, where the cultural factor means that they are chosen even in areas outside their natural habitat, which means that their area of use is greater than their ecological distribution. This is obviously also the case with those allochthonous species which are grown in this region because of long-standing traditions or cultural influences which encourage their use, be they generalized (*Allium cepa*, *Hylotelephium, Olea europea*) or more specific to a particular area (*Bidens aurea, Tanacetum parthenium, Matricaria recutita*); 2) beliefs or traditions of a particular culture, as in the case of *Tanacetum parthenium*, which is used to protect the houses in the Basque-speaking area. On the other hand, this research shows that in Navarra, cultural isolation or age-old cultural traditions are the crucial factor which keeps alive the use of particular species today (*Sideritis hyssopifolia, Anagallis*).

-The existence of numerous polysemies in Navarra have shown that one plant can replace another which is less widely available (as in the case of the chamomiles), and in many cases we were able to find out from the local names which plant was regarded as the substitute or second choice. When the use and name of two plants overlapped in one place, the plant which was considered inferior was given a pejorative name ("rough chamomile" in the case of *Santolina chamaecyparissus *y *Anthemis arvensis*, or "false tea" in the case of *Jasonia tuberosa*). However, this did not happen when two plants with different names were applied for the same purpose (as in the replacement *Hylotelephium-Anagallis*, which was clearly determined by cultural factors).

-On the other hand, the large number of species known as "chamomile" reveals a great demand for digestive medicinal plants. As the supply of the most valued ones was insufficient, less valuable species such as *Anthemis arvensis *or *Tanacetum parthenium *were used.

-Climate, substrate and culture have been shown to be useful for understanding synonymy, plant complexes or simple substitutions of plants, and we may conclude that they are very useful for discussing ethnopharmacological data.
